# Erythropoietin (*EPO*) haplotype associated with all-cause mortality in a cohort of Italian patients with Type-2 Diabetes

**DOI:** 10.1038/s41598-019-46894-2

**Published:** 2019-07-17

**Authors:** Alberto Montesano, Anna Rita Bonfigli, Maria De Luca, Paolina Crocco, Paolo Garagnani, Elena Marasco, Chiara Pirazzini, Cristina Giuliani, Fabio Romagnoli, Claudio Franceschi, Giuseppe Passarino, Roberto Testa, Fabiola Olivieri, Giuseppina Rose

**Affiliations:** 1 Department of Biology, Ecology and Earth Sciences, University of Calabria, 87036 Rende, Italy; 2 Scientific Direction, IRCCS INRCA, National Institute, Ancona, Italy; 3 Department of Nutrition Sciences, University of Alabama at Birmingham, Birmingham, AL 35294 USA; 4 Department of Experimental, Diagnostic and Specialty Medicine (DIMES), Alma Mater Studiorum, University of Bologna, Bologna, Italy; 5 Clinical Chemistry, Department of Laboratory Medicine, Karolinska Institutet at Huddinge University Hospital, Stockholm, Sweden; 6 Diabetology Unit, IRCCS INRCA, National Institute, Ancona, Italy; 7 IRCCS Istituto delle Scienze Neurologiche di Bologna, Bologna, Italy; 8 Clinical Laboratory and Molecular Diagnostics, IRCCS INRCA, Ancona, Italy; 9 Department of Clinical and Molecular Sciences, DISCLIMO, Università Politecnica delle Marche, Ancona, Italy; 10 Center of Clinical Pathology and Innovative Therapy, National Institute IRCCS INRCA, Ancona, Italy

**Keywords:** Haplotypes, Type 2 diabetes, Genetic predisposition to disease

## Abstract

Type-2 Diabetes (T2D), diabetic complications, and their clinical risk factors harbor a substantial genetic component but the genetic factors contributing to overall diabetes mortality remain unknown. Here, we examined the association between genetic variants at 21 T2D-susceptibility loci and all-cause mortality in an elderly cohort of 542 Italian diabetic patients who were followed for an average of 12.08 years. Univariate Cox regression analyses detected age, waist-to-hip ratio (WHR), glycosylated haemoglobin (HbA1c), diabetes duration, retinopathy, nephropathy, chronic kidney disease (CKD), and anaemia as predictors of all-cause mortality. When Cox proportional hazards multivariate models adjusted for these factors were run, three *erythropoietin* (*EPO*) genetic variants in linkage disequilibrium (LD) with each other (rs1617640-T/G, rs507392-T/C and rs551238-A/C) were significantly (False Discovery Rate < 0.1) associated with mortality. Haplotype multivariate analysis revealed that patients carrying the G-C-C haplotype have an increased probability of survival, while an opposite effect was observed among subjects carrying the T-T-A haplotype. Our findings provide evidence that the *EPO* gene is an independent predictor of mortality in patients with T2D. Thus, understanding the mechanisms by which the genetic variability of EPO affects the mortality of T2D patients may provide potential targets for therapeutic interventions to improve the survival of these patients.

## Introduction

T2D has an estimated prevalence of more than 9% worldwide, with the number of diabetic patients predicted to increase over the next ten years particularly due to the growth of the aging population and the increased incidence of overweight and obesity^1^. Patients with T2D have an increased risk of developing microvascular and macrovascular complications, which, in turn, are the major cause of cardiovascular deaths in these individuals^2^. Cancer as well as non-cardiovascular and non-cancer causes have also been reported to increase the risk of mortality in diabetic patients compared to nondiabetic subjects^3,4^. The underlying root factors of the relationship between diabetes complications and mortality are not fully understood and their identification is vital to improve health outcomes and reduce premature mortality rates of patients. Recently, an observational cohort study reported that modifiable lifestyle habits, such as the quality of food eaten, physical activity, and tobacco and alcohol use, are associated with reduced risk for CKD and mortality in middle-aged individuals with T2D^5^, suggesting that lifestyle behavior might in part account for this relationship. However, findings from several other observational studies argue for a more complex link between the main determinants of mortality in diabetic patients^6–9^. For instance, using data on more than 200,000 adults with T2D and after adjusting for multiple confounding factors, Kontopantelis *et al*.^8^ predicted that U-shaped relationships exist between glycated hemoglobin, blood pressure, total cholesterol, and all-cause mortality in diabetic patients. Consistently, a significant association between increased mortality and low levels of the above mentioned biological factors has also been reported in a large population of very old diabetic subjects (≥80 years of age)^9^.

T2D, diabetic complications, and their clinical risk factors have a strong genetic component^10,11^, which, in turn, might contribute to a higher risk for mortality. In this regard, previous work showed a significant association of three common genetic variants on 9p21, a chromosomal region linked to T2D and cardiovascular disease, with the mortality rate in Dutch diabetic patients^12^. Association with all-cause mortality was also reported for a variant at the glutamate-ammonia ligase (GLUL) locus, a marker of increased CHD risk^13^. Furthermore, a genetic risk score that includes 38 common T2D risk variants was found associated with mortality in an ethnic- and body weight-specific manner^14^. However, despite these findings, little is still known about the genetic factors that predispose to mortality in T2D patients. To this end, we performed a population-based, prospective cohort analysis to identify genetic risk factors for all-cause mortality in a cohort of central Italian diabetic patients, who were previously tested for genetic associations with T2D and its complications^15^.

## Results

Baseline phenotypic and clinical characteristics of the entire cohort of diabetic patients and stratified by survivors and non-survivors are shown in Table 1. The mean age of all T2D patients was 65.8 years, and 54.8% of them were men. The population had a mean diabetes duration of 15.3 years (SD 11.4 years) and on average an acceptable to good glycemic control (mean HbA1c 7.5%). At the end of an average follow-up duration of 12.08 years, 98 patients had died (18.1%). Patients who died during the follow-up were older and had significantly higher insulin resistance (HOMA-IR), serum levels of glucose, insulin, HbA1c, and average diabetes duration. Moreover, a significantly higher prevalence of nephropathy, diabetic retinopathy, and CKD characterized the patients who died during the follow-up (Table 1).

**Table 1:** Baseline characteristics of T2D patients stratified according to the occurrence of death during follow-up.

Characteristic	Total (N = 542)	Survivors (N = 444)	Non-survivors (N = 98)	P-value*
Age (yrs)	65.8 (8.0)	64.8 (7.8)	70.7 (7.3)	1.86*10^−11^
Gender (% men)	297 (54.8)	236 (53.2)	61 (62.2)	0.102
BMI (kg/m^2^)	28.9 (4.6)	28.9 (4.5)	29.0 (5.2)	0.756
WHR (mean, SD)	0.93 (0.07)	0.93 (0.07)	0.94 (0.07)	0.069
HOMA-IR (mean, SD)	2.95 (2.85)	2.79 (2.52)	3.69 (3.92)	0.032
Glucose (mean, SD) [mg/dl]	164.5 (48.8)	162.3 (47.9)	174.4 (52.1)	0.026
Insulin (mean, SD)	7.1 (5.6)	6.8 (4.9)	8.4 (8.0)	0.069
Hemoglobin (mean, SD) [mg/dl]	14.3 (1.28)	14.3 (1.62)	14.3 (1.33)	1.000
Anemia (n, %)**	22 (5.0)	11 (11.2)	33 (6.1)	0.019
RBC (mean, SD)	4.72 (0.43)	4.66 (0.52)	4.71 (0.45)	0.200
HbA1c (mean, SD) [%] [mmol/mol]	7.5 (1.3) 57.9 (13.9)	7.4 (1.3) 57.6 (13.9)	7.8 (1.3) 61.4 (13.7)	0.013
Total cholesterol (mean, SD) [mg/dl]	207.2 (37.9)	206.8 (37.2)	207.2 (40.8)	0.923
HDL cholesterol (mean, SD) [mg/dl]	52.3 (14.6)	52.5 (14.2)	51.3 (16.5)	0.505
Diabetes duration (mean, SD) [years]	15.3 (11.3)	14.06 (10.6)	20.9 (12.6)	5.0*10^−6^
Retinopathy (n, %)	152 (28.0)	111 (25.0)	41 (41.8)	0.001
Nephropathy (n, %)	73 (13.5)	52 (11.7)	21 (21.4)	0.011
Neuropathy (n, %)	101 (18.6)	76 (17.1)	25 (25.5)	0.053
Chronic Kidney Disease (n, %)	20 (3.7)	10 (2.3)	10 (10.2)	1.57*10^−4^

SD, standard deviation; BMI, Body Mass Index; WHR, waist‐to‐hip ratio; HOMA‐IR, homeostasis model assessment of insulin resistance; HbA1c, glycosylated haemoglobin; RBC, Red Blood Cells.

*t-test for continuous variables; Chi-square test for categorical variables.

**Anaemia was defined in accordance with the World Health Organization (WHO) criteria (hemoglobin levels <13 g/dL in men and <12 g/dL in women).

Overall survival rates for diabetic patients with higher values of WHR and HbA1c were worse than the other patients (Table 2). Diabetes duration also significantly influenced patient survival, with patients having a longer diabetes duration living shorter than those with shorter diabetes duration. Furthermore, diabetic patients with neuropathy nephropathy, retinopathy, CKD, and anemia lived less than patients without vascular complications (Table 2).

**Table 2:** Hazard ratios for all-cause mortality for baseline characteristics.

Variable	HR (95% CI)	P-value
Age	1.11 (1.08–1.14)	<0.001
Gender (men)	1.39 (0.92–2.09)	0.116
BMI*	1.07 (0.72–1.59)	0.750
WHR*	1.51 (1.01–2.26)	0.046
HbA1c*	1.50 (1.01–2.23)	0.045
Anaemia	2.53 (1.35–4.73)	0.004
HOMA-IR*	1.48 (0.99–2.22)	0.056
Insulin*	1.16 (0.77–1.74)	0.489
Glucose*	1.41 (0.95–2.10)	0.087
Diabetes duration (>10 yrs)	2.48 (1.51–4.09)	<0.001
Retinopathy	1.94 (1.30–2.90)	0.001
Nephropathy	1.82 (1.12–2.95)	0.015
Neuropathy	1.58 (1.00–2.49)	0.049
Chronic Kidney Disease	3.71 (1.93–7.15)	<0.001

BMI, Body Mass Index; WHR, waist‐to‐hip ratio; HbA1c, glycosylated haemoglobin; HOMA‐IR, homeostasis model assessment of insulin resistance.

*Sample mean used as a cut-off value.

**Figure 1:**
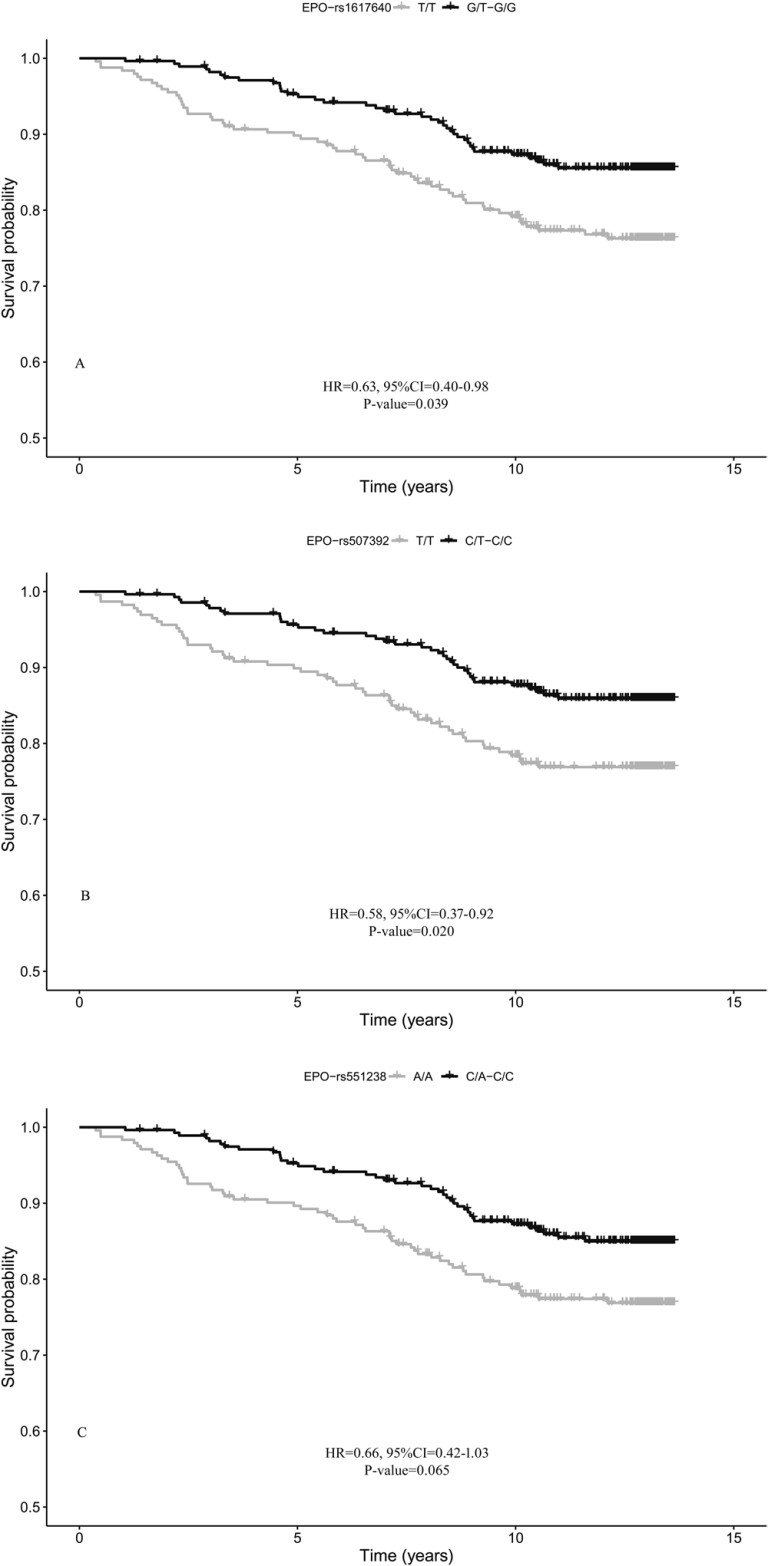
Survival functions of carriers of minor allele (black) vs non carriers (grey) of *EPO* variants. (**A**) rs1617640; (**B**) rs507392; (**C**) rs551238. Time is expressed in years, where 0 is considered the time of recruitment. The Cox regression was adjusted for age, gender WHR, HbA1c, anaemia, diabetes duration, and diabetes complications. HR value, confidence interval, and p-value from Cox regression analysis are reported inside the figure.

Kaplan–Meier survival analysis showed that the overall survival rate of diabetic patients was also correlated with five single nucleotide polymorphisms (SNPs) located in or near three different genes: rs1617640-T/G, rs507392-T/C, and rs551238-A/C in the *EPO* gene, rs1121980-C/T in the *FTO* gene, and rs1800849-C/T in the *UCP3* gene (see Supplementary Fig. S1 and Table S1). After False Discovery Rate (FDR) correction, association of *EPO*-variants with survival remained significant (q < 0.10; see Supplementary Table S1). Multivariate Cox proportional models were then fitted using age, WHR, HbA1c, diabetes duration, retinopathy, nephropathy, CKD, and anaemia as covariates. Once again, we found that variants in the *EPO* gene remained significantly associated with mortality risk at a nominal level of 0.05. As shown in Fig. 1, patients carrying the minor allele of rs1617640-(G) and rs507392-(C) had a significantly increased chance of survival with HR values of 0.63 (95% CI = 0.40–0.98, P-value = 0.039) and 0.58 (95%CI = 0.37–0.92, P-value = 0.020), respectively. There was a trend for a positive association with survival also for carriers of the minor allele of rs551238-(C) (HR = 0.66, 95%CI = 0.42–1.03, P-value = 0.065).

**Figure 2:**
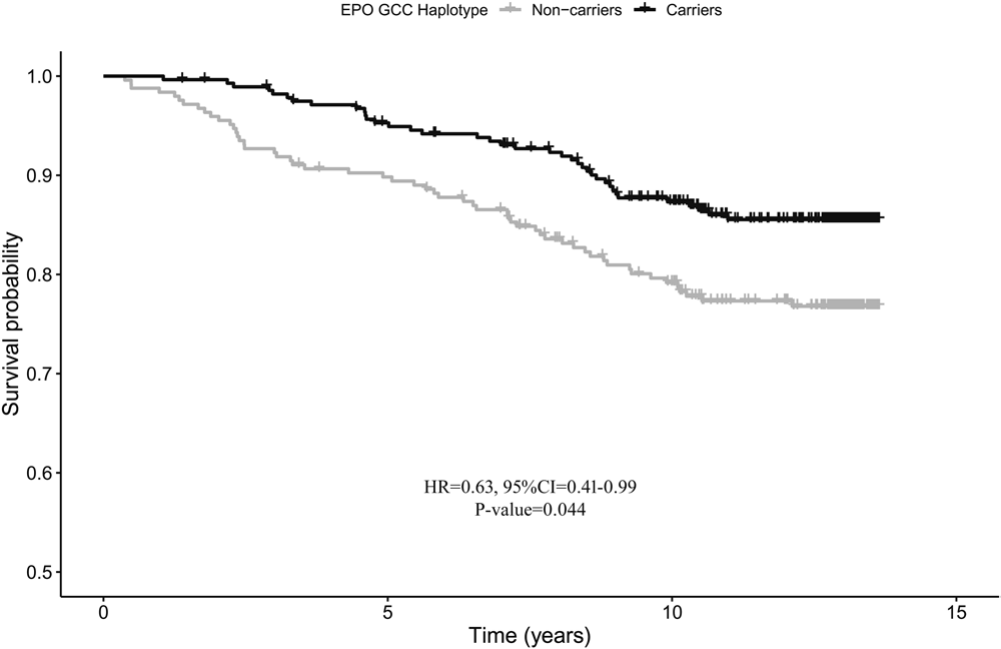
Survival functions of carriers (black) vs non carriers (grey) of the G-C-C haplotype of *EPO*. Time is expressed in years, where 0 is considered the time of recruitment. The Cox regression model was adjusted for age, gender WHR, HbA1c, anaemia, diabetes duration, and diabetes complications. HR value, confidence interval and p-value from Cox regression analysis are reported inside the figure.

LD analysis showed a high degree of disequilibrium among the three *EPO* SNPs (r^2^ > 0.97), meaning that the associations are not independent. As such, haplotype analysis was performed to further explore the relationship between *EPO* variations and diabetes mortality risk. Out of the eight possible haplotypes, two, T-T-A (major allele combination, 67.0%) and G-C-C (minor allele combination, 32.2%), were the most common in our study samples. As shown in Fig. 2, haplotype multivariate survival analysis corroborated the single-locus analyses. While the presence of the T-T-A haplotype was found to decrease the chance of survival, carriers of the G-C-C haplotype showed a significantly increased survival chance with respect to non-carriers (HR = 0.63, 95%CI = 0.41–0.99, P-value = 0.044).

## Discussion

Patients with T2D have higher all-cause mortality risk than matched individuals without diabetes^16,17^. Epidemiological studies have identified several demographic, socioeconomic, and biological independent predictors of mortality in T2D patients^8,18^, but only a few of them have evaluated the role of genetic factors. In agreement with the current literature, this study identified age, WHR, HbA1c, diabetes duration, retinopathy, nephropathy, CKD, and anaemia as predictors of all-cause mortality. Additionally, after adjusting for the effects of these risk factors, our results showed that three *EPO* genetic variants in high LD (rs1617640-T/G, rs507392-T/C and rs551238-A/C) were associated with all-cause mortality risk in both single-SNP and haplotype-based analyses.

The *EPO* gene encodes erythropoietin, which is a kidney-derived cytokine that plays a major role in promoting erythropoiesis, in particular in response to hypoxic stress^19^. However, recent evidence shows that *EPO* is also expressed locally in others tissues and organs, including peripheral endothelial cells, muscle, and insulin-producing cells, where it displays anti-apoptotic, anti-inflammatory, and angiogenic effects due to its ability to activate several transduction signalling pathways^20–23^. A growing literature argues for a critical role of EPO in diabetes and its complications^24^. For example, Fenjves *et al*. demonstrated that *EPO* overexpression in human islet cells provides protection from cell death^25^. Studies in model organisms also suggest that *EPO* can protect against diabetes through a direct effect on β cells^26^, and possibly through the modulation of glucose metabolism, glucose tolerance, and insulin sensitivity^27^. Furthermore, Abhary and colleagues identified three genetic variants in *EPO* associated with a higher risk to develop diabetic retinopathy^28^. Although no significant associations between *EPO* SNPs and T2D or its vascular complications were identified in our earlier analysis^15^, the findings in this study corroborate the close and complex link between erythropoietin and diabetes status.

A weakness of this study is that the specific causes of death were not determined during follow-up, preventing us from conducting an in-depth evaluation of the association of the investigated SNPs with the mortality risk in diabetic patients. Also, the mechanism through which the *EPO* TTA haplotype increases mortality in T2D patients is unknown at this time. However, it is important to point out that Tong and colleagues previously found that the *EPO* rs1617640-T allele is associated with a 25-fold higher promoter activity compared with the G allele, suggesting that this SNP plays a significant functional role in *EPO* expression^29^. Taken together with our results this observation suggests that high levels of erythropoietin may have detrimental effects in patients with T2D. This idea is supported by several pieces of evidence. Elevated erythropoietin concentrations have been linked to proliferative diabetic retinopathy, which in turn is associated with excessive vascular growth^30^. A study by Wagner and colleagues also showed that in diabetic patients with CKD, elevated endogenous EPO levels were predictive of all-cause mortality and related mainly to markers of inflammation independently of kidney function and haemoglobin levels^31^. Similar results were observed in people aged 85 years and older^32^ and in patients with heart failure^33,34^. Given that our analysis did not detect any correlation between the studied *EPO* SNPs and the presence/absence of anaemia (data not shown), it is plausible that inflammation, a key emerging risk factor for T2D, is a potential mechanism linking EPO and mortality in T2D patients.

It has been shown that EPO acts as a proliferative factor and thereby can promote tumor growth and metastasis^35^. Ongoing studies indicate that the use of EPO for the treatment of cancer-induced anaemia is related to an increased incidence of cancer progression and reduced survival of patients^36^. Epidemiologic evidence indicates that diabetes is associated with increased risk of many types of cancer^37^; therefore, it is also possible that higher levels of EPO might contribute to the increased risk of mortality in diabetic patients because of an increased incidence of cancer. In this regard, it is notable that an *EPO* polymorphism (rs576236) in LD with those here investigated confers susceptibility to adrenal tumor^38^.

In conclusion, this study identified an *EPO* haplotype that increases the risk of mortality among patients with T2D, independently of non-genetic risk factors. Over the last 30 years, it has become clear that erythropoietin possesses several tissue-specific functions that go far beyond the regulation of red cell production; therefore, further studies are needed to elucidate the molecular mechanisms linking EPO and mortality in diabetic patients. The elucidation of the mechanisms underlying the observed genetic associations could not only enhance our understanding of the molecular and genetic basis of the disease but also provide valuable insights into potential targets for therapeutic interventions aimed at increasing the survival of diabetic patients.

## Methods

### Study population and genetic data

Classification criteria and details of the cohort as well as the genetic data used in this study were previously reported^15^. Briefly, the sample includes 542 individuals with T2D (mean age 65.8 ± 8.0), collected by the Diabology Unit, INRCA (National Institute on Health and Science on Aging) in Ancona (Italy). Briefly, demographic, anthropometric and clinical data were collected for each individual and recorded in a well-defined questionnaire. The presence/absence of diabetic complications was evidenced as follows: diabetic retinopathy by fundoscopy through dilated pupils and/or fluorescence angiography; incipient nephropathy, defined as an urinary albumin excretion rate > 30 mg/24 h and a normal creatinine clearance; CKD, defined as an estimated glomerular filtration rate <60 mL/min per 1.73 m^2^; neuropathy was established by electromyography.

Ethical approval for this study has been granted by the Ethics Committee of National Institute on Health and Science on Aging (INRCA). All participants gave written informed consent. All methods were performed in accordance with the relevant guidelines and regulations.

The genetic data includes 40 SNPs located in or near 21 candidate genes. However, nine SNPs were excluded from the analysis because they had either a high proportion of missing genotypes (call rate lower than 90%; rs669173, rs2853669, rs4880, rs13266634, rs7901695, rs8047395) or a minor allele frequency (MAF) lower than 5% (rs3811791, rs16889462, rs2237892). The final dataset included 31 high-quality SNPs that were tested for association with survival (see Supplementary Table S1).

### Outcome

The main outcome of our study was all-cause mortality. The average follow-up duration was 12.08 years (range 1.41–13.66 years). At each follow-up visit, information was collected on vital status, functional status, and occurrence of diabetes complications. For patients who died during the follow-up period, information about date and place of death were collected from death certificates provided by relatives or caregivers. City or town registers were consulted to retrieve information about death when death certificates were not provided. After this period 61 men (20.5%) and 37 women (15.1%) died.

### Analytic approach

Unpaired t-test and chi-square were performed for continuous and categorical variables, respectively, to compare variables and covariates between dead and survived patients at the end of the follow-up period. Cox regression models were used to evaluate the effect of anthropometric and haematological parameters, anaemia, diabetes duration, and diabetes complications on mortality. The length of survival from baseline visit until death was used as failure time for the models. Survivors were censored on the day of the last follow-up visit. The proportional hazard assumption was checked graphically, plotting the log-minus-log survival function over time. Kaplan-Meier analysis was performed to estimate survival curves stratified for each SNP. To control for the false-positive rate, the FDR method was used; the cut-off of the FDR adjusted p-value (q-value) was 0.10.

Covariates significantly associated with survival (p < 0.05) in the univariate analyses were included as confounding factors in the multivariate Cox proportional hazards models. These models were used to identify SNPs/haplotypes significantly associated with all-cause mortality. In all analyses, genetic data were coded using a dominant model (carriers for the minor allele versus non-carriers).

Pairwise measures of LD between the analyzed loci were calculated with the Haploview 4.2^39^. The amount of LD was quantified by Lewontin’s coefficient (D’).

Statistical analyses were performed using the survival package of R v3.4.2 statistical language (R Foundation for Statistical Computing, Vienna, Austria, www.r-project.org).

## Supplementary information

Supplementary Information

## Data Availability

The dataset analysed during the current study is available from the corresponding author on reasonable request.
