# Author Correction: Erythropoietin (*EPO*) haplotype associated with all-cause mortality in a cohort of Italian patients with Type-2 Diabetes

**DOI:** 10.1038/s41598-020-59859-7

**Published:** 2020-02-21

**Authors:** Alberto Montesanto, Anna Rita Bonfigli, Maria De Luca, Paolina Crocco, Paolo Garagnani, Elena Marasco, Chiara Pirazzini, Cristina Giuliani, Fabio Romagnoli, Claudio Franceschi, Giuseppe Passarino, Roberto Testa, Fabiola Olivieri, Giuseppina Rose

**Affiliations:** 1 Department of Biology, Ecology and Earth Sciences, University of Calabria, 87036 Rende, Italy; 2 Scientific Direction, IRCCS INRCA, National Institute, Ancona, Italy; 3 Department of Nutrition Sciences, University of Alabama at Birmingham, Birmingham, AL 35294 USA; 4 Department of Experimental, Diagnostic and Specialty Medicine (DIMES), Alma Mater Studiorum, University of Bologna, Bologna, Italy; 5 Clinical Chemistry, Department of Laboratory Medicine, Karolinska Institutet at Huddinge University Hospital, Stockholm, Sweden; 6 Diabetology Unit, IRCCS INRCA, National Institute, Ancona, Italy; 7 IRCCS Istituto delle Scienze Neurologiche di Bologna, Bologna, Italy; 8 Clinical Laboratory and Molecular Diagnostics, IRCCS INRCA, Ancona, Italy; 9 Department of Clinical and Molecular Sciences, DISCLIMO, Università Politecnica delle Marche, Ancona, Italy; 10 Center of Clinical Pathology and Innovative Therapy, National Institute IRCCS INRCA, Ancona, Italy

Correction to: *Scientific Reports* 10.1038/s41598-019-46894-2, published online 17 July 2019

The original version of this Article contained a typographical error in the spelling of the author Alberto Montesanto, which was incorrectly given as Alberto Montesano. This has now been corrected in the PDF and HTML versions of the Article, and in the accompanying Supplementary Information file.

