# Supplementary material for: Erythropoietin (EPO) haplotype associated with all-cause mortality in a cohort of Italian patients with Type-2 Diabetes
Source: Sci Rep. 2019 Jul 17;9:10395. doi: 10.1038/s41598-019-46894-2 (PMC6637129; doi:10.1038/s41598-019-46894-2)
Supplement: Supplementary file 1 — Supplementary Information [file 41598_2019_46894_MOESM1_ESM.pdf]

Erythropoietin (*EPO*) haplotype associated with all-cause mortality in a cohort of Italian patients with Type-2 Diabetes

Alberto Montesano, Anna Rita Bonfigli, Maria De Luca, Paolina Crocco, Paolo Garagnani, Elena Marasco, Chiara Pirazzini, Cristina Giuliani, Fabio Romagnoli, Claudio Franceschi, Giuseppe Passarino, Roberto Testa, Fabiola Olivieri, Giuseppina Rose

Table S1: List of the analyzed polymorphisms and their association with T2D survival

Gene	CHR	SNP	Major/Minor allele	MAF	Chi-square*	p-value	q-value**
DDAH1	1	rs13373844	A/C	0.337	1.131	0.287	0.619
DDAH1	1	rs7521189	A/G	0.457	0.209	0.647	0.799
IRS1 (500 kb-3'UTR	2	rs2943641	C/T	0.399	0.341	0.558	0.744
ADIPOQ	3	rs266729	C/G	0.248	0.122	0.726	0.804
ADIPOQ-AS1	3	rs1063539	G/C	0.107	0.526	0.468	0.716
IGF2BP2	3	rs1470579	A/C	0.355	0.371	0.542	0.744
IGF2BP2	3	rs4402960	G/T	0.364	1.031	0.309	0.619
TERC	3	rs12696304	C/G	0.273	0.318	0.572	0.744
UCP1	4	rs3811787	T/G	0.258	3.599	0.057	0.250
UCP1	4	rs45539933	C/T	0.064	0.099	0.752	0.804
UCP1	4	rs1800592	A/G	0.239	1.476	0.224	0.632
CLPTM1L	5	rs401681	C/T	0.414	0.524	0.468	0.716
TERT	5	rs2735940	T/C	0.411	1.349	0.245	0.619
TERT	5	rs2736098	G/A	0.298	0.780	0.377	0.700
TERT	5	rs2736109	G/A	0.414	0.149	0.699	0.804
VEGFA	6	rs3025021	C/T	0.332	0.093	0.760	0.804
EPO	7	rs1617640	T/G	0.322	7.696	0.005	0.072
EPO	7	rs507392	T/C	0.330	7.670	0.005	0.072
EPO	7	rs551238	A/C	0.324	6.417	0.011	0.097
TCF7L2	10	rs7903146	C/T	0.446	2.620	0.105	0.342
CAT	11	rs1001179	G/A	0.215	0.595	0.440	0.716
KCNJ11	11	rs5215	T/C	0.360	0.061	0.804	0.804
UCP2	11	rs660339	C/T	0.342	2.783	0.095	0.342
UCP3	11	rs1800849	C/T	0.166	5.261	0.021	0.141
HIF1A	14	rs11549465	C/T	0.146	0.072	0.787	0.804
FTO	16	rs8050136	C/A	0.473	1.573	0.209	0.605
FTO	16	rs9939609	T/A	0.468	1.035	0.308	0.619
FTO	16	rs1121980	T/C	0.489	3.953	0.046	0.243
FTO	16	rs1421085	T/C	0.487	0.700	0.402	0.702
FTO	16	rs17817449	T/G	0.472	1.341	0.246	0.632
APOE	19	rs440446	G/C	0.399	0.259	0.610	0.755

*Chi-square value for the log-rank test

** False discovery rate (FDR) P-values adjusted for the multiple comparisons (cut-off =0.10).

MAF: minor allele frequency

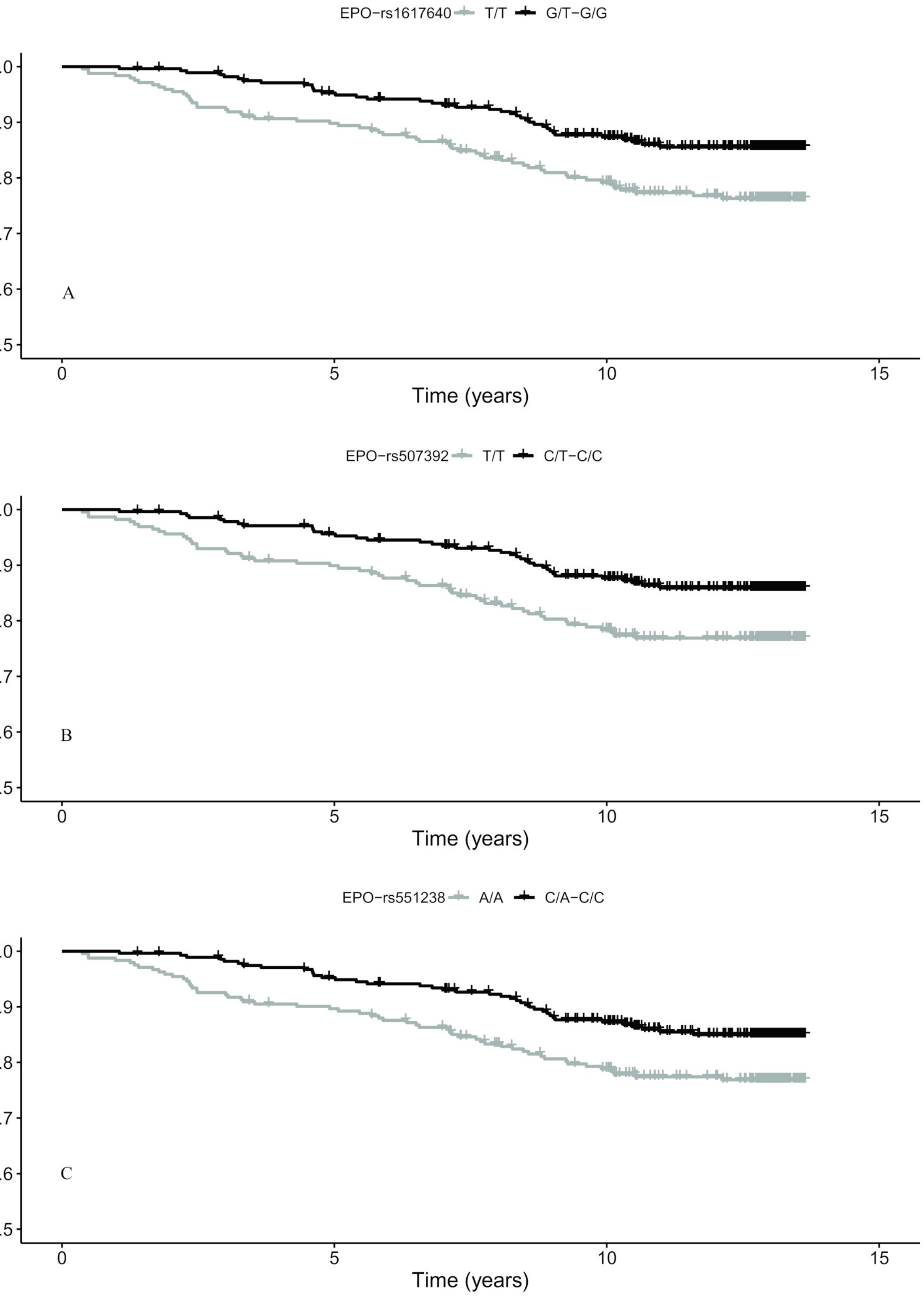

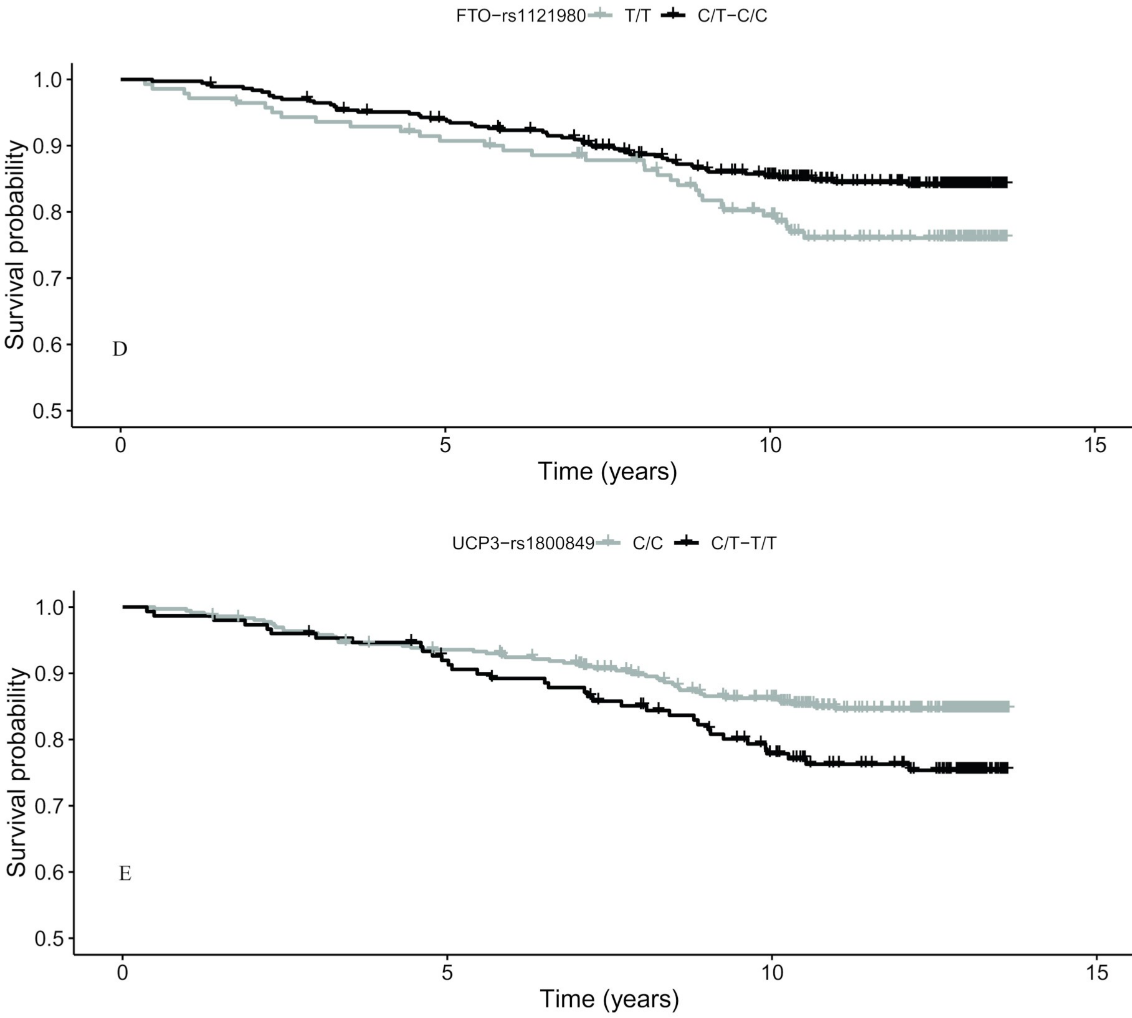

Supplementary Figure S1.
Kaplan–Meier survival analysis of T2D patients stratified according to carriers of minor allele (black) vs non carriers (grey) of EPO (A-C), FTO (D), UCP3 (E) variants.
Time is expressed in years, where 0 is considered the time of recruitment.
